# Effects of Oral Topical Capsaicin Gel on Taste Perception in Healthy Subjects: A Pilot Study

**DOI:** 10.1111/jop.13620

**Published:** 2025-03-17

**Authors:** Katia Rupel, Alex Buoite Stella, Martina Tamos, Daniela Adamo, Federica Canfora, Matteo Biasotto, Roberto Di Lenarda, Giulia Ottaviani

**Affiliations:** ^1^ Department of Medicine, Surgery and Health Sciences University of Trieste Trieste Italy; ^2^ Department of Neuroscience, Reproductive Sciences and Dentistry University of Naples Federico II Naples Italy

**Keywords:** capsaicin gel, dysgeusia, gustatory sensation, taste strips

## Abstract

**Objectives:**

Topical capsaicin is widely used for managing peripheral neuropathies; however, its impact on gustatory perception following prolonged oral use remains unclear. This pilot study aimed to evaluate changes in gustatory sensitivity and food preferences induced by capsaicin topical gel therapy in healthy individuals.

**Materials and Methods:**

Ten healthy female subjects applied capsaicin gel (0.025%) to the gingival mucosa twice daily for 14 days. Evaluations were conducted at baseline (T0), after 2 weeks (T1), after 4 weeks (T2), and after 4 weeks following discontinuation (T3). A matched control group underwent identical assessment without capsaicin application. Gustatory changes were measured using a modified taste strip method and a food preferences questionnaire.

**Results:**

While subjective alterations in food perception, liking, and preferences were reported in the capsaicin group, no significant objective changes in gustatory perception (intensity and recognition of salty, sweet, sour, and bitter flavors) were observed. Subjective changes were reversible upon cessation of capsaicin use.

**Conclusions:**

Topical capsaicin gel influences subjective food perception and preferences without objectively altering gustatory function. These findings highlight the importance of considering such effects when prescribing capsaicin for oral somatosensory disorders, such as burning mouth syndrome or dysgeusia.

## 1 Introduction

Orofacial sensory disturbances, including altered somatosensory and gustatory sensitivity, play a significant role in the pathogenesis of chronic idiopathic orofacial pain syndromes [1, 2, 3]. These conditions are often accompanied by dysgeusia, a distorted taste perception, which can significantly impact food preferences, nutrition, and overall quality of life [4, 5].

Emerging evidence suggests that receptors from the transient receptor potential (TRP) and G protein‐coupled receptor (GPCR) families are involved in the modulation of orofacial sensory function [6, 7]. Capsaicin, a well‐characterized TRPV1 agonist, induces prolonged receptor activation, leading to desensitization and reduced nociceptive signaling [8]. This mechanism underlies the therapeutic use of capsaicin for peripheral neuropathies [9]. Our recent work demonstrated significant changes in thermal sensory and pain thresholds following repeated capsaicin application in the oral cavity [10].

Despite the established somatosensory effects of capsaicin, its influence on taste perception and food preferences remains poorly understood. This pilot study investigates whether repeated topical capsaicin application induces changes in gustatory sensitivity, flavor recognition, and food preferences in healthy individuals.

## 2 Materials and Methods

### 2.1 Study Design and Population

This prospective, case–control study adhered to the Declaration of Helsinki and was approved by the University of Trieste Ethics Committee (protocol number 134_2023). Twenty healthy female participants aged ≥ 18 years were enrolled. Inclusion criteria were limited to female gender to minimize gender‐related variability in taste perception. Exclusion criteria included dysgeusia, prior capsaicin exposure, habitual consumption of spicy food, and medication use.

Participants were randomly assigned to either the capsaicin (CAP) group (*n* = 10) or the control (CTRL) group (*n* = 10). CAP subjects applied 0.025% capsaicin gel twice daily for 4 weeks, while CTRL subjects underwent no intervention. All participants were instructed to avoid spicy food during the study period. Evaluations were performed at baseline (T0, before starting the application of the gel), 2 weeks (T1), 4 weeks (T2), and after 4 weeks after discontinuation (T3).

### 2.2 Capsaicin Gel

Consistent with previous studies [11, 12], participants in the CAP group received 0.025% capsaicin gel in 10 mL syringes. The gel was prepared as a compounded medication with the following composition (for 50 g): capsaicin 0.0125 g, 95° alcohol 2.5 g, hyaluronic acid 0.75 g, glycerin 5 g, sodium benzoate 0.05 g, purified water 41.5875 g. The gel was applied twice daily to the gingival mucosa of the anterior maxilla (regions 12/13) and mandible (regions 42/43). Participants refrained from brushing, eating, drinking, or chewing for at least 30 min before and after application.

### 2.3 Gustatory Testing

Taste perception was evaluated using a modified “Taste Strips” protocol [13]. The gustatory testing consists of placing on the tongue 4 filter paper disks (dimensions 9.5 × 9.5 mm each) impregnated with solutions representing one of the four main flavors: salty (150 mM NaCl), sweet (300 mM saccharose), sour (6 mM citric acid), and bitter (0.05 mM quinine hydrochloride). Participants identified each taste and rated intensity on a Numerical Rating Scale (NRS, 0–10).

### 2.4 Questionnaire

Subjective changes in food perception, liking, and preferences were assessed via a structured questionnaire administered at T1, T2, and T3 (Data S1).

### 2.5 Statistical Analysis

Statistical analyses were conducted using Prism (v9.1.0, GraphPad Software) and R (v4.0.2). Data normality was assessed using the Shapiro–Wilk test. Repeated‐measures ANOVA and mixed‐effects ANOVA were employed for intra‐ and inter‐group comparisons, with post hoc Tukey correction. Chi‐square tests were used for ordinal variables. A *p*‐value < 0.05 was considered statistically significant.

## 3 Results

Twenty participants (CAP: *n* = 10, mean age 21 ± 2 years; CTRL: *n* = 10, mean age 22 ± 2 years) completed the study, with no significant age differences between groups.

### 3.1 Taste Intensity

In the CAP group, the intensity of each flavor did not vary significantly over time (from T0 to T3) (Salty: *p* = 0.52; Sweet: *p* = 0.12; Bitter: *p* = 0.28; Sour: *p* = 0.43). In the CTRL group, the perceived intensity of all the flavors analyzed did not vary significantly (Salty: *p* = 0.24; Sweet: *p* = 0.57; Bitter: *p* = 0.75; Sour: *p* = 0.26).

**FIGURE 1:**
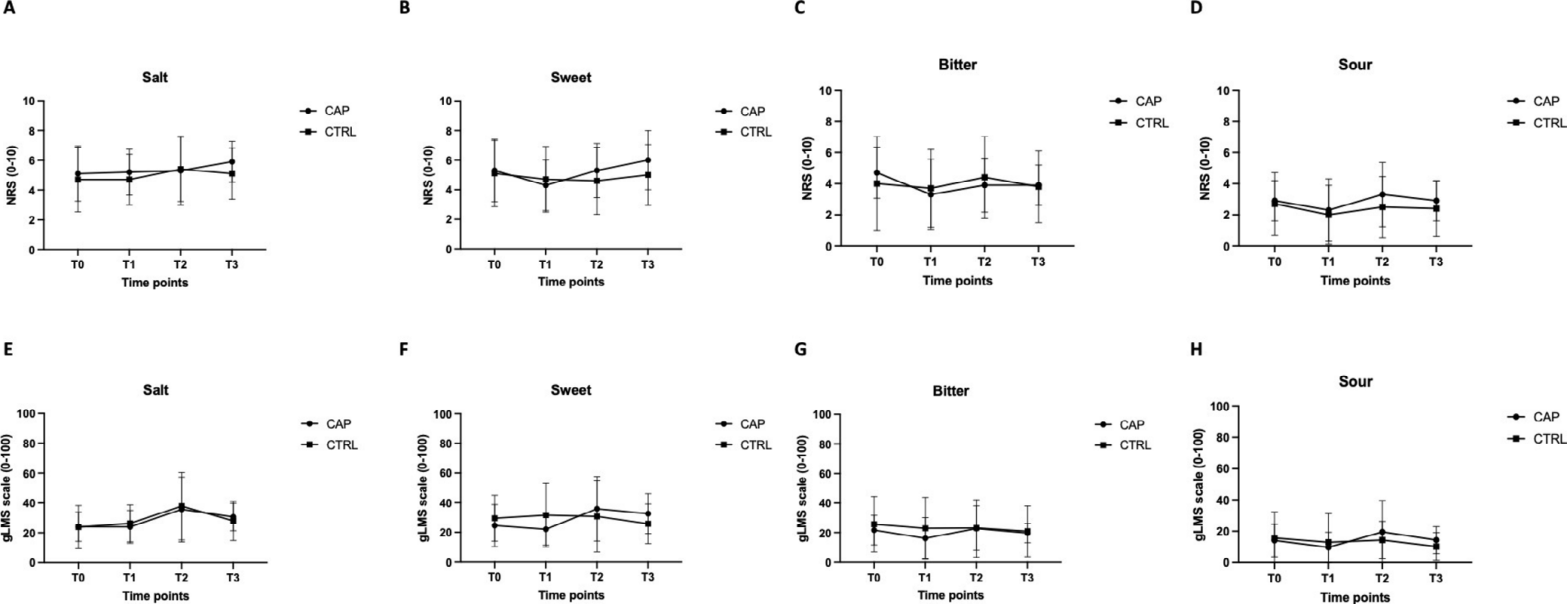
Taste intensity testing over time in CAP and CTRL groups, evaluated using the NRS (0–10) scale and gLMS (0–100) scale. (A, E) salty flavor. (B, F) sweet flavor. (C, G) bitter flavor. (D, H) sour flavor. Data are plotted as means ± standard deviations (error bars).

No significant interactions were found when analyzing the group × time effect (Salty: *p* = 0.66; Sweet: *p* = 0.36; Bitter: *p* = 0.73; Sour: *p* = 0.84). Therefore, no significant differences between CAP and CTRL groups were found in the intensity of each taste over the experimental time points. Data are represented in Figure 1.

### 3.2 Taste Recognition

No significant difference was found in flavor recognition at individual time points between the two groups (CAP vs. CTRL Pearson's chi‐squared test *p* = NS at T0, T1, T2, T3 for each taste type) or within each group over time (from T0 to T3 for each taste type). Data including exact *p*‐values are reported in Table 1.

**TABLE 1:** Taste recognition capacity over time in CAP and CTRL groups. Data are reported as a percentage of the total (*n* = 10 subjects for each group).

	Salty					Sweet				
	T0	T1	T2	T3	*p* ^a^	T0	T1	T2	T3	*p* ^a^
CAP	100%	90%	80%	100%	0.27	100%	100%	90%	100%	0.38
CTRL	100%	100%	100%	100%	1	100%	100%	100%	100%	1
*p*‐value^a^	1	0.31	0.14	1		1	1	0.31	1	

	Bitter					Sour				
	T0	T1	T2	T3	*p* ^a^	T0	T1	T2	T3	*p* ^a^
CAP	80%	70%	70%	80%	0.91	80%	80%	70%	90%	0.74
CTRL	100%	90%	90%	100%	0.55	90%	80%	80%	90%	0.88
*p*‐value^a^	0.12	0.26	0.26	0.14		0.53	1	0.61	1	

Abbreviations: CAP, capsaicin group; CTRL, control group; NS, not significant.

^a^ Pearson's chi‐squared test.

Subsequently, we evaluated possible differences in the proportions of subjects who did not recognize at least one type of flavor at every time point and found no significant differences between the CAP and CTRL groups at any time point.

### 3.3 Food Perception, Liking and Preferences

**FIGURE 2:**
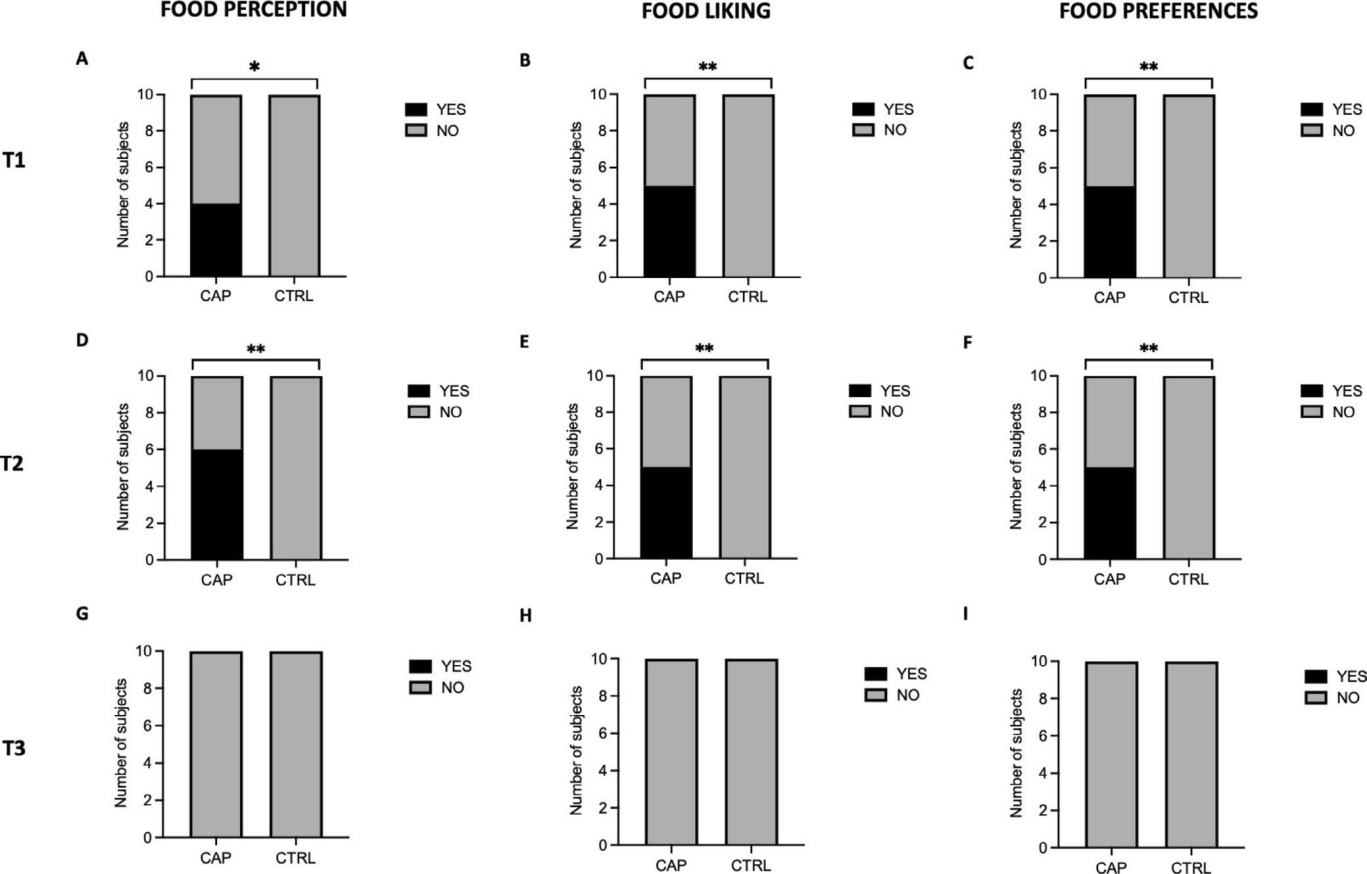
Food perception, liking, and preferences from T1 to T3. (A, D, G) changes in food perception at T1, T2, and T3. (B, E, H) changes in food liking at T1, T2, and T3. C,F,**I** changes in food preferences at T1, T2, and T3 **Pearson's chi‐squared test *p* < 0.01. *Pearson's chi‐squared test *p* < 0.05. Data are reported as the number of subjects.

Results of the questionnaires showed a significant difference between the CAP and CTRL groups at T1 in liking (*p* = 0.01), perception (*p* = 0.03), and preferences (*p* = 0.01) of food and drinks, as represented in Figure 2A–C. At T2, there was an increase in the subjects that reported alterations in food perception in the CAP group, so the significant difference between groups was maintained (*p* = 0.003), as shown in Figure 2D. Significant differences in food liking and preferences were stable from T1 to T2 (*p* = 0.01 for both variables), as represented in Figure 2E,F.

At T3, no subjects reported any residual alteration, and consequently, there were no differences between groups in any of the variables (*p* = 1 for all variables), as shown in Figure 2G–I.

Notably, in the CTRL group, there was no change in the perception, liking, or preferences of foods and drinks at any time point.

40% of subjects using capsaicin gel experienced an increase in the perception of the sweet and salty flavors of foods at T1 and T2. The perception of all flavors increased in 20% of capsaicin users. 50% of CAP subjects reported variations in food liking and specifically a decrease in liking for sweets and an increase in liking for spicy foods. From T1 to T2, the intensity of the variations further increased. All reported changes did not persist following the cessation of use of the gel (at T3).

## 4 Discussion

This study provides preliminary evidence that topical capsaicin gel influences subjective food perception and preferences without impacting objective gustatory function. As regards the primary flavors examined (salty, sweet, bitter, and sour), the participants who used the gel containing capsaicin did not show variations in the perceived intensities during the entire period of use of the gel (T0 to T2) and after its discontinuation (T3), without significant differences when compared to the control group. Additionally, the results also revealed that the use of a capsaicin‐based gel did not alter the recognition of flavors over time and after its cessation, also when compared to a control group.

These results are consistent with the fact that spiciness is not considered a sense of taste, and no taste receptor cells for this specific sensation in the taste buds have been identified yet [14]. Spiciness is considered a multimodal trigeminal stimulation [15], often associated with pain and heat, initiated by the activation of TRPV1 channels [16].

However, the influence of spicy compounds on gustatory function and perception of foods has been previously described. While our results are in line with the study by Cowart [17], they are in contrast with the results of other studies [18], according to which the perceived intensities of all flavors decrease after rinsing with a solution containing capsaicin. However, these studies determined the effect of capsaicin on flavor intensity immediately after intake, but not after repeated use.

Results of questionnaires showed that the use of the capsaicin‐based gel provoked significant subjective alterations in the perception of foods/drinks, in food liking, and preferences. Capsaicin users experienced an increase in the perception of sweet and salty flavors, with a reduced preference for sweets and an increase in liking for spicy foods. However, it was a reversible phenomenon that regressed completely within 4 weeks after discontinuing the use of capsaicin gel.

It was suggested [19] that the more frequent the intake of capsaicin, the lower the intensity of the perceived burning, suggesting a possible explanation for the increased liking of spicy foods. However, the decrease in liking of sweets reported by the participants is in disagreement with other studies [20, 21].

Several authors suggested that capsaicin use might be beneficial for the treatment of oral somatosensory disorders, including burning mouth syndrome, because of its analgesic effects [22, 23]. Indeed, these clinical entities are often of complicated management and can also be associated with dysgeusia, ultimately influencing food preferences and nutrition [4, 5]. The significant variations that emerge from this study suggest that the influence of topical capsaicin use on food perception and preferences should be considered when evaluating the effect of topical capsaicin on oral somatosensory disorders.

## 5 Conclusions

Topical capsaicin gel induces transient subjective changes in food perception and preferences, without objectively altering gustatory function. However, this is a reversible phenomenon that regressed completely within 4 weeks after discontinuing the use of capsaicin gel. These findings underscore the importance of monitoring sensory changes during capsaicin therapy in oral disorders.

## Author Contributions

**Katia Rupel:** conceptualization, supervision, formal analysis, writing – original draft preparation; **Alex Buoite Stella:** conceptualization, supervision, writing – review and editing; **Martina Tamos:** investigation; **Daniela Adamo and Federica Canfora:** methodology, formal analysis; **Matteo Biasotto:** supervision, methodology, writing – review and editing; **Roberto Di Lenarda:** supervision, validation; **Giulia Ottaviani:** supervision, validation, visualization.

## Ethics Statement

All the participants were requested to sign an informed consent. All procedures were approved by the ethical committee of the University of Trieste (protocol code 134/2023, 09.2023).

## Conflicts of Interest

The authors declare no conflicts of interest.

### Peer Review

The peer review history for this article is available at https://www.webofscience.com/api/gateway/wos/peer‐review/10.1111/jop.13620.

## Supporting information


**Data S1.** Supporting Information.

## Data Availability

The data that support the findings of this study are available from the corresponding author upon reasonable request.
