# Supplementary material for: Effects of Oral Topical Capsaicin Gel on Taste Perception in Healthy Subjects: A Pilot Study
Source: J Oral Pathol Med. 2025 Mar 17;54(5):392–6. doi: 10.1111/jop.13620 (PMC12077945; doi:10.1111/jop.13620)
Supplement: Supplementary file 1 — Data S1. Supporting Information. [file JOP-54-392-s001.docx]

**Supplementary File 1**

**Questionnaire**

1) Have you noticed any changes in the perception of the taste of foods and drinks since you started using the gel that was delivered to you?

☐ Never ☐ Rarely ☐ Sometimes ☐ Often ☐ Always

If you answered "Never" to the previous question, go directly to question 10, otherwise continue with question 2.

2) Have you noticed any changes in the BITTER taste?

☐ Yes ☐ No ☐ I don't perceive this taste

3) If you answered “Yes”, how has your perception of the BITTER taste changed?

☐ Reduction of taste perception ☐ Increase of taste perception

4) Have you noticed any changes in the SWEET taste?

☐ Yes ☐ No ☐ I don't perceive this taste

5) If you answered “Yes”, how has your perception of the SWEET taste changed?

☐ Reduction of taste perception ☐ Increase of taste perception

6) Have you noticed any changes in the SOUR taste?

☐ Yes ☐ No ☐ I don't perceive this taste

7) If you answered “Yes”, how has your perception of the SOUR taste changed?

☐ Reduction of taste perception ☐ Increase of taste perception

8) Have you noticed any changes in the SALT taste?

☐ Yes ☐ No ☐ I don't perceive this taste

9) If you answered “Yes”, how has your perception of the SALT taste changed?

☐ Reduction of taste perception ☐ Increase of taste perception

10) Have you noticed a change in your eating preferences for certain foods?

☐ Never ☐ Rarely ☐ Sometimes ☐ Often ☐ Always

11) Have you noticed a change in your liking of certain foods?

☐ Never ☐ Rarely ☐ Sometimes ☐ Often ☐ Always

If you answered from "Little" to "Very Much" please answer the following questions, if you answered "Nothing" do not no longer has to answer any questions.

12) What foods do you like least since you started using the gel that was delivered to you? You can select multiple response alternatives.

☐ Fruits

☐ Sweets

☐ Pasta/Rice/Bread

☐ Meat/Fish

☐ Raw vegetables

☐ Cooked vegetables

☐ Other (please specify):________________________

13) What foods do you like most since you started using the gel that was delivered to you? You can select multiple response alternatives.

☐ Fruits

☐ Sweets

☐ Pasta/Rice/Bread

☐ Meat/Fish

☐ Raw vegetables

☐ Cooked vegetables

☐ Other (please specify):________________________
